# “The educating nursing staff effectively (TENSE) study”: design of a cluster randomized controlled trial

**DOI:** 10.1186/s12912-014-0046-6

**Published:** 2014-12-19

**Authors:** Theo J G M Hazelhof, Debby L Gerritsen, Lisette Schoonhoven, Raymond T C M Koopmans

**Affiliations:** Vitalis WoonZorggroep Eindhoven, Eindhoven, the Netherlands; Department of Primary and Community Care: Centre for Family Medicine, Geriatric Care and Public Health, Radboud University Medical Centre, Nijmegen, the Netherlands; Scientific Institute for Quality of Healthcare (IQ healthcare), Radboud University Medical Center, Nijmegen, the Netherlands; Faculty of Health Science, University of Southampton, Southampton, UK; Joachim en Anna, Centre for Specialized Geriatric Care, Nijmegen, the Netherlands

**Keywords:** Dementia, Challenging behaviour, Training, Nursing staff, Stress, Work satisfaction

## Abstract

**Background:**

Challenging behavior exhibited by people with dementia can have adverse outcomes, like stress, low morale, low work satisfaction and absenteeism for nursing staff in long-term care settings. Training nursing staff to manage challenging behavior may reduce its impact. Although much of the research into training nursing staff shows methodological limitations, several studies find some effect of training programs on knowledge about and on management of challenging behavior. Effects on stress or burnout are almost not found.

**Methods/Design:**

The TENSE-study is a randomized controlled study on 18 nursing home units (9 control, 9 intervention) investigating the effects of a continuous educational program for nursing staff about managing challenging behavior. Nursing staff of intervention units receive the program, nursing staff of control units do not and continue usual care. The primary outcome is stress experienced by nursing staff (N = 135). Secondary outcomes are: emotional workload, work satisfaction, stress reactions at work and knowledge about challenging behaviour of nursing staff; and frequency of challenging behavior, quality of life and social engagement of residents (N = 135). Because there are many unknown factors influencing the effect of the training, a process evaluation to evaluate sampling-, implementation- and intervention quality as well as barriers and facilitators to implementation will also be included in the analysis. Nursing staff could not be blinded to the intervention, but were blinded for the outcomes.

**Discussion:**

Strengths of this study are the (good) description of the intervention complemented by a process evaluation and the tailoring of the intervention to the wishes and needs of the nursing homes at any moment during the study. Sustaining the effects of the intervention by using follow up sessions is another strength. Possible drawbacks may be dropout because of the frailty of the elderly population and because nursing staff might move to another job during the study.

**Trial registration:**

NTR (Dutch Trial Registration) number NTR3620

## Background

Working with residents with dementia is enervating for nursing staff. Up to 97% of nursing home residents with dementia exhibit challenging behavior during the course of the dementia [1]. Challenging behavior is associated with adverse outcomes in nursing staff, such as anxiety, less work satisfaction, emotional exhaustion [2,3], physical health stress and burnout [4]. Agitated behavior, and physically aggressive behavior in particular, is strongly related to distress in nursing staff, especially if this behavior is perceived as threatening [4-6]. In addition, if the cause of the behavior is unclear [7] or if nursing staff anticipates aggressive behavior [7,8] and consider themselves not adequately equipped to manage the behavior, they are prone to experiencing stress [9,10]. Stress can lead to low morale, absenteeism from work and high staff turnover [4]. It can also negatively affect the interaction with residents and thus the quality of provided care [11]. Decreasing adverse outcomes of challenging behavior for nursing staff may be accomplished by training them to better manage the behavior [12].

Many studies into educational programs for nursing staff in long term care settings have very small sample sizes, non random designs, designs without control groups or a low response rate [13,14]. Only a minority of these programs is evaluated in an RCT-design.

Studies of the effect of educational programs for nurses regarding challenging behavior generally focus on knowledge [15-24]; management skills to decrease or cope with challenging behavior[19,24-31]; stress experienced by nurses [16,17,19,24,26,29,32,33] and/or resident behavior [2,19,21,24-26,28,34-36].

Although most studies that focused on knowledge found an increase in knowledge [15,17-24,37], there is minimal evidence that the increased rates of knowledge can be sustained [13]. Only one of the three available RCT’s [17,19,21] reports on follow up meetings and found an increase in knowledge post intervention at three and six months follow up [19]. Follow up sessions to update knowledge are highly recommended by several authors [14,22,25,29,31,32,38].

Studies that focused on management skills resulted in (significant) improvements in different nursing skills. [18,24-29,31]. Three of these studies, two RCT’s [25,26] and one using a quasi-experimental pre- and posttest design [31], succeeded in sustaining these improvements.

Of the studies that focused on stress, one RCT [32] and two quasi experimental pre- and post design studies [16,29], found a small effect: The RCT [32] found an increase in stress in the control group but not in the intervention group which was given a training followed by making individual care plans and supervision of a psychiatric nurse. One quasi experimental study [16] found that nursing staff viewed the task of care giving as less frustrating and more rewarding, the other [29] found that after the intervention nursing staff showed fewer stress. Two RCT’s [17,19] found no effect.

Of the studies that focused on challenging behavior the non-RCT studies [2,19,26-28,36] found no differences in challenging behavior of the residents. Three RCT’s [21,34,35] and a controlled study [25], found an effect of training programs for nurses regarding challenging behavior on the behavior of the residents. Two of those [34,35] found a significant decrease in the total agitation scores as measured by the CMAI, and two [21,34] found significant decreases on physically non-aggressive behavior and on verbally aggressive behavior. Only one study [21] found a significant effect on aggressive behavior, which was also measured by the CMAI.

In summary, studies show effects on knowledge. However, there is little evidence for sustaining these effects in the long term. In general, studies that focused on management skills found an effect. A sustained effect was found on management skills and resident behavior outcomes for some of the training programs that use a kind of follow up. There are three studies that found lasting effects on outcomes for nursing staff as well as outcomes for residents [21,25,34].

Almost no effects on stress were found. In general, there are only a few high quality studies that investigated the long term effect of the interventions [19,25,26,29,31,32]. Thus, more than a decade after the conclusion of Aylward et al. [14] that there is a lack of rigorous research into the effectiveness of continuous education programs in long term care, this lack still exists.

Besides the quality of the studies, there is also a problem with the description of the educational programs. These were often poorly or not described so we are unable to judge the quality of these programs. Some of the studied programs did not combine knowledge with skills training [11,15,16]. Furthermore, because of the lack of description of the follow up sessions, it is not possible to evaluate whether the programs were tailored enough. An educational program has to support various learning styles of the participants [39-41]. Studies about learning styles of nurses found that (student) nurses have a mix of all learning styles [39,40], but that more than half have a predominantly concrete learning style and almost half a reflective learning style [42]. This implies that training generally has to be interactive and multifaceted [39-41].

An important factor needed for sustaining the results is integrating the educational program in daily practice by tailoring it to individual needs and the needs and interest of the care-organization [37]. We hypothesize that implementation of an educational program that does not have the aforementioned shortcomings will more likely result in a decrease of distress experienced by the nursing staff and a decrease in frequency of challenging behavior. Therefore, we developed an educational program for nursing staff on challenging behavior that is tailored to the wishes and needs of the care-organization and combines various learning styles.

The aim of our study is to determine, in a randomized design that accounts for clustering within units, the effect of a new educational program in the short and in the mid- term on stress experienced by nursing staff (primary outcome), emotional workload, work satisfaction, stress reactions at work and knowledge about the origin and the management of challenging behavior of nursing staff and challenging behavior of the residents. We will also determine the effect of the program on Quality of Life (QoL) and social engagement of residents as stress and work satisfaction may influence the way residents are treated by nursing staff. Possibly intervening factors such as attitude about people with dementia, the organizational culture, the time residents have lived on the unit and the time nursing staff works on the unit are measured as well.

## Methods

### Design

The TENSE-study is a cluster-randomized, controlled study on dementia special care units (DSCU) of Dutch nursing homes from different regions of the Netherlands. Nursing homes will participate after consent of the management, the unit managers, nursing staff, the board of representatives of residents and the psychologist. Eighteen units (clusters) will be randomized with a block size of 2. From each of 9 nursing home organizations, two units are included, one of which becomes an intervention unit (receives a training program) and one becomes a control unit (no training, continuing usual care). This results in nine intervention units and nine control units. Allocation to intervention or control was determined through the tossing of a coin by an independent researcher unfamiliar with the study and blinded for the nursing homes. To prevent contamination, the nursing homes need to agree that the control and the intervention unit do not have the same physician and psychologist and that nursing staff members are not employed at both units during the study period. Units are preferably at a different location.

**Figure 1:**
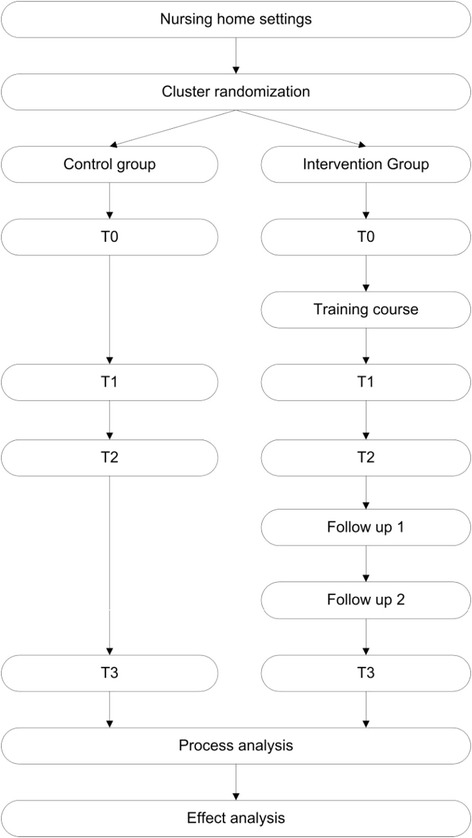
**Design of the TENSE-study.**

The duration of the follow-up will be 9 months. A 3-day course (three times 2,5 hours) for intervention units will be held immediately after baseline (T0), with follow up sessions after three and six months. Assessments will take place at baseline (T0), immediately after the course (T1), just before follow up session 1 (T2), and nine months after the 3 day course (T3). Table 1 gives an overview of the measurements (see Figure 1).

**Table 1:** **Design of the study, time schedule**

**Outcomes**	**Month:**	**1**	**2**	**3**	**4**	**5**	**6**	**7**	**8**	**9**	**10**	**11**
		T0	I, T1			T2, F1			F2			T3
**Nursing staff**	**Primary**											
	Emotional Burnout: (UBOS)	X	X			X						X
	**Secondary**											
	Emotional workload (NPI-NH)	X				X						X
	Work satisfaction (LAKS)	X				X						X
	Emotional reactions during work (subscale VBBA)	X				X						X
	Knowledge	X	X									X
**Residents**	**Secondary**											
	Challenging behavior (NPI-NH)	X				X						X
	Agitation (CMAI)	X				X						X
	Quality of life: (Qualidem)	X				X						X
	Social Engagement (RISE)	X				X						X
	Perceived Quality of care (Self-developed)	X				X						X
Additional measurements												
	Approaches towards dementia (ADQ)	X				X						X
	Organizational culture (CVF)	X				X						X

I = Intervention (training program).

T = Time of measurement.

F = Follow up session of training program.

UBOS = Utrecht Burnout Scale.

NPI-NH = Neuro Psychiatric Inventory Nursing Home.

CMAI = Cohen Mansfield Agitation Index.

Qualidem = Quality of life of residents with dementia in nursing homes.

RISE = Revised Index for Social Engagement for long-term care.

ADQ = Approaches towards Dementia Questionaire.

CVF = Competing Values Framework of organizational culture.

VBBA = Vragenlijst Beleving en Beoordeling van de Arbeid.

### Inclusion criteria

Nursing homes will be included if:

- they have at least two DSC Units;
- nursing staff members, psychologist and physician work on one of these units exclusively;
- one care team cares for at least fifteen residents that are diagnosed with dementia;
- participating units have not planned a reorganization or other interventions that can influence the study within half a year before or during the study.

Inclusion criteria for residents are:

- dementia according to DSM-IV-TR criteria [43],
- living in the nursing home for more than one month.

Inclusion criteria for the nursing staff are:

- being employed for at least three months and not expected to be transferred to another unit within the study period,
- working more than one day a week.

### The intervention

The educational program has three core elements for increasing its usefulness:

1. an individually tailored make up based on interviewing staff, management and nursing staff about their wishes and needs;
2. two follow up sessions, to enable continuous education;
3. supervision, monitoring, facilitation and stimulation by the psychologist, middle management and other staff on the use of the newly learned techniques.

It has the following content:

- providing knowledge about dementia,
- educating about the origins of challenging behavior and how to manage it,
- educating how to recognize distress caused by challenging behavior in yourself and in colleagues,
- practicing how to gather information on resident and environmental factors that cause challenging
- behavior,
- practicing how to signal signs of impending challenging behavior,
- practicing general behavior management skills that can be used in approaching the resident,
- practicing how to use a standardized method to manage challenging behavior, which is integrated in the multidisciplinary work processes and forms that are used in the nursing home.

The content of the educational program is based on three conceptual models regarding challenging behavior: (1) The Progressively Lowered Stress Threshold Theory (PLST) [44], (2) the Unmet Needs [45] model and (3) the ABC model [46-48]. These three theories are used to explain possible antecedents for different forms of challenging behavior. The PLST (1) states that residents with dementia are less able to cope with stress as they get tired during the day. Unmet Needs theory (2) claims that challenging behavior is caused by the decreased capability of clients with dementia to explain their needs. The ABC model (3) is used for analyzing the antecedents and consequences of behavior. It is developed by Cohn et al. [22], and introduced in the Netherlands by Hamer en Voesten [46,47]. The ABC model is based on learning theory [49] and states that behavior (B) is triggered by internal and external stimuli, (Antecedents (A)) that bring the resident to this behavior. Behavior is reinforced by Consequences (C). In this program the ‘D’ is added to the ABC model, regarding thoughts and feelings of nursing staff about the challenging behavior. (in Dutch this is the “D” from “Denken” which means: “think”). The ABCD model is used for describing challenging behavior. Participants gather extensive information about antecedents in the past (personality, important life events and coping style), the present (cognitive- and communication capacities), the environment (the unit, other residents and the nursing staff) and the family (interaction with the resident).

A pilot program/training was run with one group of fifteen nurses. This training course was judged as very good by the participants and led to a few minor adjustments of the program (using more case studies brought in by the participants). The training program was performed by one trainer.

### The process of implementation

Before the 3-day course starts an extensive semi structured interview is conducted by the trainer with the psychologist, the elderly care physician (ECP) [50], the team manager and some members of the nursing staff to explore current (multidisciplinary) work processes and collect wishes and needs of the institution for tailoring the program. In this interview the following topics are discussed (1) in what way does the actual way of working differ from an ideal situation? (2) the context for change; is the nursing staff motivated to change?, (3) in what way does the organization supply help to facilitate changes in work processes. (4) what barriers and facilitators for the process of change exist in the nursing home. After the interview a plan is presented to the care-organization which describes in what way the intervention is tailored to the care-organization by training the elements that need to be changed [51]. The researcher receives the forms used by nursing staff on the unit for observing and analyzing behavior and for making a behavioral management plan, and incorporates these forms into the educational program. This way, nursing staff will not be confronted with new forms which differ from the ones they use in daily practice. The training program was provided by a very experienced professional trainer of nursing staff.

#### The course

The 3-day course is offered to the complete unit-team and consists of three lessons of 2.5 hours, provided once per week or, once every two weeks. During the course, several team members are made responsible for various tasks, such as observing the resident’s behavior; observing stress caused by the challenging behavior; making an action plan; controlling the use of the plan; and involving the physician and/or psychologist at the right moment. Furthermore, the unit managers also receive the training so they can facilitate and reward the use of the newly learned techniques. Two follow up sessions are carried out. These are designed to stimulate the process of managing challenging behavior methodologically and are tailored to the situation in the organization.

#### First session: causes of challenging behavior and consequences for the resident. Introducing the ABCD model to analyze challenging behavior

Knowledge about dementia and causes for challenging behavior are explained. Several theories explaining challenging behavior are used, including the Unmet Needs theory and the Progressive Lowered stress Threshold Theory (PLST). Using the ABCD model participants also analyze their own role in causing and (accidentally) rewarding challenging behavior. They receive a form with which they can describe a resident that exhibits challenging behavior. These cases are used in the second session.

#### Second session: consequences of challenging behavior for nursing staff

Participants bring in two or more cases from their unit. These are used to analyze challenging behavior. The information they have gathered is used to answer the next five questions; (A) what unmet need is the resident expressing with this challenging behavior? (B) How does the resident cope with (a lower) stress (threshold) now and difficulties given his cognitive and verbal capacities. (C) What is the influence of the environment on the clients behavior? (D) What is the influence of the nursing staff on the challenging behavior? (E) What is the influence of the family of the resident on the challenging behavior? In doing this, it becomes clear for nursing staff on which part they need more information.

#### Third session: a plan of action

Before the third session nursing staff gathers the information they need and also read a paper about making a plan of action [52]. The third session concerns making the action plan. Nursing staff learn to use the sequence of “problem – goal – action” to decide what action has to be taken. Nursing staff decides which aspects of the training they want to practice in the next three months.

Subsequently, the trainer writes a report about the training that consists of the way participants want to manage the challenging behavior after the training and the wishes for future training and sends it to the psychologist of the nursing home. The psychologist, the unit manager and the elderly care physician can use it to support the nursing staff in managing challenging behavior and incorporate what they have learned in daily practice.

#### Follow-up sessions

There is a follow up session at three and six months after the 3-day course. These follow up sessions are tailored to the situation in the nursing home after interviewing the unit manager, the psychologist and the nursing staff about the implementation of the acquired knowledge in the training course, their experiences and the subsequent training they still need. If possible the trainer joins in a regular team meeting. Follow up sessions are all designed to stimulate the process of managing challenging behavior methodologically but can train different aspects in each nursing home.

Tailoring the training course to suit it to the unit is done by applying two follow up meetings in which parts of the training program that need additional attention are addressed [53]. Furthermore, qualitative information on current multidisciplinary care processes and the level of knowledge of nursing staff on the individual units is collected before the start of the training program. This information is used to determine how the program and the newly acquired knowledge can be integrated in the unit’s multidisciplinary care process, and to determine whether the program’s content must be preceded by additional information to increase the participants’ initial level of knowledge.

### Outcome measures

Nursing staff fills in questionnaires with questions about themselves and the residents at four points in time on the outcome measures described below. To prevent information bias, nursing staff are not informed about the scores [54]. Data is collected using a secured web based questionnaire (www.tense-studie.nl).

#### Primary outcome measure

Stress is operationalized using the Dutch version of the Maslach Burnout Inventory (Maslach and Jackson, [55]), the ‘Utrecht Burnout Scale – C’ (Schaufeli and van Dierendonck, [56]). This scale measures three components of burnout: emotional exhaustion, depersonalization and decreased personal accomplishment. Higher scores on this six-point scale suggest higher stress [57]. Internal consistency is good with Cronbach’s alpha of .70 and over [57], and the validity of the three factor structure has been confirmed [57].

#### Secondary outcome measures

##### Nursing staff

To measure emotional workload, the subscale ‘emotional workload’ of the Neuro Psychiatric Inventory Nursing Home version is used (for a description of the NPI NH: see below).

*Work satisfaction* is measured with the Dutch “Leiden Quality of work Questionnaire”. The Leiden Quality of Work Questionnaire scale consists of seven subscales: completeness of the job, organizational tasks, cycle length, complexity, autonomy, possibilities for social contact, and feedback. These seven scales have a satisfactory internal reliability [58].

*Stress reactions* at work are assessed with the subscale” emotional reactions at work” from the: “Vragenlijst Beleving en Beoordeling van de Arbeid” (VBBA). This four-point scale can be considered as uni-dimensional, and reliability and validity are good [59].

A *knowledge-test* based on knowledge tests found in literature [60-70] is used before and after the educational program, and at the final measurement, this test was run in a pilot with 25 nurses in a nursing home.

##### Residents

*Challenging behavior* is assessed using the Neuro Psychiatric Inventory-Nursing home (NPI-NH) and the Cohen-Mansfield Agitation Inventory (CMAI). The NPI-NH: a comprehensive assessment scale including the following symptoms: delusions, hallucinations, agitation, depression, anxiety, euphoria, apathy, disinhibition, irritability, aberrant motor behavior, night-time disturbances and eating change. The frequency (F) is rated on a four-point (1–4) Likert scale and the severity (S) is rated on a three-point (1–3) Likert scale, yielding an F X S score. When a symptom is not present, the F and S scores are both zero. The F X S score thus contains information about prevalence, frequency, and severity (range 0–12 for each symptom). A Dutch translation of the NPI has also shown to be reliable and valid [49].

The Cohen-Mansfield Agitation Inventory (CMAI) consists of 29 items about agitation and aggression and has been validated for use in care homes in the Netherlands [71]. The CMAI measures the frequency (on a seven point scale from never to several times an hour) of agitation during the preceding two weeks (total score range: 29–203). The NPI-NH and the CMAI are commonly used in the nursing home so nursing staff is used to these forms.

For measuring *Quality of life* the Qualidem is used. The Qualidem includes 37 items and is a multidimensional scale specifically designed for institutionalized residents with dementia. The Qualidem is designed by Ettema et al. [72,73] and evaluated by Bouman [74]. It measures the quality of life for people with dementia living in residential care settings. It assesses quality of life of all residents even those with severe dementia [74]. It consists of nine subscales: care relationship, positive affect, negative affect, restless tense behavior, positive self image, social relation, social isolation, feeling at home and having something to do.

Social engagement will be measured by the Revised Index for Social Engagement (RISE) developed by Gerritsen et al. [75] and based on the Index for Social Engagement (Mor et al., [76]). This scale describes social engagement for residents living in long-term care. Reported internal consistency and interrater and intrarater reliability are sufficient [75].

#### Additional measurements

As it has been shown that attitudes of nursing staff about dementia and on the other hand organizational culture may influence the extent to which innovations are executed or may influence the strength of the intervention effect, possible influencing factors and their association with the primary and secondary outcomes will be investigated. To this aim the following scales are included in the study:

##### Approaches about people with dementia

To measure nursing staff attitudes, the Approaches towards Dementia Questionnaire (ADQ: Lintern & Woods, [77]) is assessed. This scale consists of 19 statements about people with dementia measured on a five point Likert scale. This scale has two subscales, one indicates the staff member’s degree of hopefulness and the other indicates the extent to which a person-centered approach is exposed. The subscales have shown good reliability and have been validated against direct observation of the quality of staff care interactions [78].

##### Organizational culture

Organizational culture will be measured with the Competing Values Framework of organizational culture (CVF) for long-term care as developed by Scott-Cawiezell et al. [79]. The CVF assesses the 6 dimensions of the competing values framework in 6 items. These are: dominant organizational characteristic, administration, management style, organizational glue, strategic emphasis and criteria for success [80].

### Process evaluation

Along with the intervention study a process evaluation is carried out according to the model of Leontjevas et al. [81]. It is important to study whether the educational program was delivered as intended to draw accurate conclusions on its effects (Hulscher et al., [51]). Also, process evaluation enables improvement of the intervention, enables others to replicate the program, facilitates future comparison between studies (Hulscher et al., [51]) and enables the transition from research evidence into health practice (Grol and Grimshaw, [82]).

To study the reach of and compliance to the program, attendance to sessions of the nursing staff is registered. Dropout of residents by death or relocation and replacement of them are registered. To register the feasibility and relevance of the training program nursing staff is asked to evaluate the training. Barriers and facilitators for the implementation of the changes nursing staff is willing to do are gathered. At the end of the study ECP’s, unit managers and psychologists will be asked in a semi-structured interview if they have perceived changes in the management of challenging behavior on the unit.

### Sample size

For calculating the appropriate sample size we use the following assumptions:

DSCU consist of 20 residents on average [49], with 18 nursing staff on average eligible for inclusion.

For the primary outcome, stress experienced by nursing staff, we assume that our intervention leads to a 4 point decrease on the subscale Emotional Exhaustion of the Maslach Burnout Inventory based on the reduction found by Yun-Hee Jeon et al. [83] who found a reduction of 2.5 points after three months and 4.4 points after nine months in her study in which she used training and support to evaluate person centred care to dementia care mapping and usual care. Based on these assumptions and based on a significance level alpha of 0.05, a power of 0.80 and a conservative estimated correlation between two measurements of .6 [84] and an ICC of 0.05 we need a sample size of 121 nursing staff members and 121 residents in each group for ANCOVA analysis based on equal sample sizes which implies including 16 clusters; eight intervention and eight control units. In order to account for intra cluster correlations, Multi Level Analysis will be used. Nursing staff that moves to another job will be replaced, the nursing staff replacing them will join in the course. To compensate for nursing staff not replaced in time we will add an extra unit so we use nine intervention and nine control units this results in 135 nursing staff members and 135 residents in the intervention group and an equal amount in the control group.

### Ethical approval

The study is undertaken in accordance with the declaration of Helsinki (http://www.wma.net/en/30publications/10policies/b3/index.html), the applicable Dutch legislation and in agreement with the Conduct Health Research (version 2005; http://www.federa.org/gedragscodes-codes-conduct-en). It has been assessed by the Medical Ethics Committee of the region Arnhem-Nijmegen in the Netherlands. According to Dutch legislation and the committee, the study can be carried out without a review procedure by the committee because residents are not actively involved in data collection for the study, all resident data consist of observations made by the nursing staff. In addition, approval is asked from the local ethics committees of participating Nursing Homes.

Legal representatives are informed about the study and the aim to include the resident in the study, and are given the opportunity to refuse. Representatives are also informed that they can withdraw their relative at any moment in the study. Nursing staff provided informed consent before filling in the questionnaires on the website.

### Data analysis

Statistical analysis will be performed using the Statistical Package for Social Sciences (SPSS). The UBOS score will be used as primary outcome and emotional workload, work satisfaction, stress reactions at work and knowledge about challenging behavior as secondary outcomes for nursing staff in an ANCOVA analysis in which outcome score is the score after intervention controlling for the score before the intervention. Age, sex and time working on the unit will be used as covariates. The NPI-NH and the CMAI will be used as outcomes for the residents. Age, sex and length of stay will be used as covariates. For the primary and secondary outcome analyses, multilevel linear regression and multilevel logistic regression analyses on units, individual nurses and residents will be used [85]. These analyses will calculate effects on distress (NPI, UBOS), challenging behavior (NPI-NH, CMAI), quality of life of residents (Qualidem, RISE), and perceived quality of care.

## Discussion

The TENSE-study is a cluster randomized controlled study investigating the effects of a continuous educational program on stress experienced by nursing staff (primary outcome) and emotional workload, work satisfaction, stress reactions at work and knowledge about challenging behaviour; and frequency of challenging behavior, quality of life and social engagement of residents. Client outcomes are secondary outcomes in this study. Although client outcomes are very important and have also been subject of earlier research into training programs for nursing staff, we do realize that to see an effect on client outcomes there probably has to be an effect on the outcomes for nursing staff first.

Strengths of this study are: the elaborate description of the intervention and the process evaluation in which factors that influence the study will be described. Strengths of the intervention are: tailoring the intervention to the wishes and needs of the care-organization to overcome barriers and profit from facilitators in the care-organization. The use of follow up sessions to sustain the results is positive as well. Other strengths of the intervention are the contact between the trainer and the nursing home that allows us to adjust the training program at every moment in time.

The chosen design is suitable for our purposes. It allows us to measure effects of the training program on stress and knowledge directly after the training, and the ‘short’ (3 months) and mid- term effects (9 months) by performing two additional measurements. We expect that the tailor made follow up meetings will enhance sustainability of the training results. To increase the motivation of the nursing staff, the program will be delivered to the control units when the study is finished.

The outcome measures are all commonly used in the nursing homes and they have been shown to be suitable for residents with dementia and for nursing staff. This study is one of the first nursing home studies that gathers its data by using a specifically designed website. The study has some weaknesses that should be mentioned. One limitation is that nursing staff will be aware of receiving the intervention, which may cause bias. To limit this bias, nursing staff will not be informed about the scores on the outcome measures. Another drawback is the frailty of the research population, so that the proportion of residents that dies during the research can be significant. Replacing the deceased residents may be difficult. The sample size is adjusted for the proportion of nursing staff that moves to another job. A final limitation of this study is that we are not able to study the results longer than nine months.
